# Targeted delivery of doxorubicin by CSA-binding nanoparticles for choriocarcinoma treatment

**DOI:** 10.1080/10717544.2018.1435750

**Published:** 2018-02-09

**Authors:** Baozhen Zhang, Guogang Cheng, Mingbin Zheng, Jinyu Han, Baobei Wang, Mengxia Li, Jie Chen, Tianxia Xiao, Jian Zhang, Lintao Cai, Shoujun Li, Xiujun Fan

**Affiliations:** a Laboratory for Reproductive Health, Shenzhen Institutes of Advanced Technology, Chinese Academy of Sciences, Shenzhen, China;; b Guangdong Key Laboratory of Nanomedicine, CAS Key Lab for Health Informatics, Institute of Biomedicine and Biotechnology, Shenzhen Institutes of Advanced Technology, Chinese Academy of Sciences, Shenzhen, China;; c Guangdong Provincial Key Laboratory of Prevention and Control for Severe Clinical Animal Diseases, Guangzhou, China;; d College of Veterinary Medicine, South China Agricultural University, Guangzhou, China

**Keywords:** Chondroitin sulfate A, CSA-bind peptide, nanoparticles, drug delivery, tumor targeting, choriocarcinoma

## Abstract

Gestational trophoblastic neoplasia (GTN) can result from the over-proliferation of trophoblasts. Treatment of choriocarcinoma, the most aggressive GTN, currently requires high doses of systemic chemotherapeutic agents, which result in indiscriminate drug distribution and severe toxicity. To overcome these disadvantages and enhance the chemotherapeutic efficacy, chondroitin sulfate A (CSA)-binding nanoparticles were developed for the targeted delivery of doxorubicin (DOX) to choriocarcinoma cells using a synthetic CSA-binding peptide (CSA-BP), derived from malarial protein, which specifically binds to the CSA exclusively expressed in the placental trophoblast. CSA-BP-conjugated nanoparticles rapidly bonded to choriocarcinoma (JEG3) cells and were efficiently internalized into the lysosomes. Moreover, CSA-BP modification significantly increased the anti-cancer activity of the DOX-loaded nanoparticles *in vitro*. Intravenous injections of CSA-BP-conjugated nanoparticles loaded with indocyanine green (CSA-INPs) were rapidly localized to the tumor. The CSA-targeted nanoparticles loaded with DOX (CSA-DNPs) strongly inhibited primary tumor growth and, more importantly, significantly suppressed metastasis *in vivo*. Collectively, our results highlight the potential of the CSA-BP-decorated nanoparticles as an alternative targeted delivery system of chemotherapeutic agents for treating choriocarcinoma and for developing new GTN therapies based on drug targeting.

## Introduction

Gestational trophoblastic neoplasia (GTN) causes malignant lesions arising from the over-proliferation of placental trophoblasts and includes invasive moles, choriocarcinoma, placental site trophoblastic tumors, and epithelioid trophoblastic tumors (Lurain, 2011; Nadhan et al., 2017). Gestational choriocarcinoma, the most aggressive gestational trophoblastic disease, develops in approximately one in every 50,000 pregnancies (Seckl et al., 2010) and triggers massive local trophoblast invasion and vascular permeation, leading to metastasis to the brain, kidney, liver, and lung (Shih, 2007; Cheung et al., 2009). The serum concentrations of human chorionic gonadotropin (hCG) increase in advanced stages of choriocarcinoma and are useful for diagnosing choriocarcinoma and for monitoring the effects of chemotherapy on the disease progression (Kohorn, 2001). Depending on the Federation of Gynecology and Obstetrics (FIGO) score of choriocarcinoma, the first-line treatment is either single agent systemic chemotherapy or a polychemotherapy regimen of methotrexate, etoposide, actinomycin D, cyclophosphamide, and vincristine (Taşçı et al., 2016; Frost et al., 2017). However, the systemic administration of chemotherapeutic agents results in indiscriminate drug distribution and severe toxicity.

Nanomedicine is a multidisciplinary research field that merges concepts from medicine and nanotechnology with the overall goal of accurately fine-tuning the biological processes occurring at the micron and submicron scales (Ferrari, 2005; Peer et al., 2007; Riehemann et al., 2009). One of the great advantages of nanomedicine over traditional molecular therapeutics is the ability to vector drugs preferentially to the affected loci, thus increasing drug efficacy and reducing associated adverse reactions. Our collaborative team has developed a lipid–polymer nanoparticle platform that has been adapted to simultaneously deliver high-dose chemotherapy and photothermal agents to tumor regions in a breast cancer xenograft mouse model without having adverse effects (Zheng et al., 2013; Zhao et al., 2014).

Another important recent advance has been the clarification of the key event underlying placental malaria pathogenesis and the concurrent discovery of the malarial protein VAR2CSA. Specifically, the malaria parasite *Plasmodium falciparum* replicates within infected erythrocytes (IEs), which effectively sequester in the placental intervillous spaces of a pregnant woman. Placental adherence is mediated by the malarial VAR2CSA protein exposed on the IE membrane (Salanti et al., 2004; Gamain et al., 2005; Duffy et al., 2006). This protein binds a distinct type of chondroitin sulfate A (CSA) that is exclusively expressed in placental syncytiotrophoblasts (Fried & Duffy, 1996; Salanti et al., 2003, 2004, 2015). Notably, the minimal CS binding region of VAR2CSA consists of the Duffy binding ligand-like (DBL) 2X domain with flanking interdomain (ID) regions (Dahlbäck et al., 2011; Clausen et al., 2012). Furthermore, the binding of this domain to CS was confirmed using a synthetic CSA-binding peptide (CSA-BP) (Resende et al., 2008).

Inspired by the new insights into placental malaria, we hypothesized that the CSA-BP could be used as a tool for the targeted delivery of chemotherapeutic agent-loaded nanoparticles to placental trophoblasts to treat GTN, particularly choriocarcinoma. In the present study, we investigated the targeted choriocarcinoma efficacy of CSA-targeted nanoparticles *in vitro*, *in vivo*, and *ex vivo.* Moreover, the efficacy of the CSA-targeted nanoparticles loaded with doxorubicin (CSA-DNPs) in alleviating the primary tumor burden and inhibiting choriocarcinoma metastasis was studied by administering the CSA-DNPs to tumor-bearing mice following multiple intravenous injections.

## Materials and methods

### Chemicals

Soybean lecithin and 1,2-distearoyl-*sn*-glycero-3-phosphoethanolamine-N-[carboxy (polyethylene glycol)-2000] (DSPE-PEG-COOH) were purchased from Avanti Polar Lipids (Alabaster, AL). Poly(dl-lactic-*co*-glycolic acid) (50:50) (PLGA), 1-ethyl-3-(3-dimethylaminopropyl) carbodiimide hydrochloride (EDC) and N-hydroxysuccinimide (NHS), doxorubicin (DOX) and indocyanine green (ICG) were purchased from Sigma-Aldrich (St. Louis, MO). CSA-binding peptide (EDVKDINFDTKEKFLAGCLIVSFHEGKC) (Resende et al., 2008) and the scrambled peptide (SCR, EVDNDKKLGLVFEKDKIFTEFACISHCG) were synthesized by ChinaPeptides Co., Ltd. (Shanghai, China) and Shanghai GL Biochem Co. Ltd. (Shanghai, China), respectively. Unless stated otherwise, all other materials used were obtained from Sigma-Aldrich (St. Louis, MO).

### Cell lines and transfection

JEG3 cells were obtained from the Cell Bank of the Chinese Academy of Sciences (Shanghai, China) and cultured in DMEM/F12 (Gibco, Grand Island, NY) supplemented with 10% fetal bovine serum (FBS) (Gibco, Grand Island, NY). To produce the firefly luciferase (Fluc)-GFP-positive JEG3 cell line (Fluc-GFP-JEG3), lentiviruses expressing the Fluc and GFP fusion protein (LV-Fluc-GFP) were produced as described previously (Fan et al., 2011) and then were used to transduce the JEG3 cells. The LV-Fluc-GFP-transduced JEG3 cells were sorted for pure Fluc-GFP-positive JEG3 cells using BD FACSAria III (BD Biosciences, San Jose, CA).

### Preparation of DNPs and INPs

DOX-loaded nanoparticles (DNPs) or ICG-loaded nanoparticles (INPs) were synthesized from PLGA, soybean lecithin, DOX or ICG, and DSPE-PEG-COOH using the single-step sonication method (Zheng et al., 2013). PLGA was dissolved in acetonitrile with a concentration of 2 mg/mL. Stock solutions of soybean lecithin and DSPE-PEG-COOH were dissolved separately in 4% ethanol aqueous solutions with concentrations of 1 mg/mL, and DOX or ICG was prepared at a concentration of 10 mg/mL in ultrapure water. To prepare the DNPs, 750 μg of DOX, 90 μg of soybean lecithin and 210 μg of DSPE-PEG-COOH were added to 3 mL of a 4% ethanol aqueous solution, and then, 1 mL PLGA acetonitrile solution was added dropwise under sonication using an ultrasonic processor (VCX130, Newtown, CT) at a frequency of 20 kHz and a power of 130 W for 5 min. The DNPs were then concentrated by ultracentrifugation (34,000×*g*) (Optima TM MAX-XP, Beckman, Brea, CA) at 4 °C for 30 min. After discarding the supernatant, the samples were re-suspended in 1 mL of phosphate-buffered saline (PBS, pH7.4). Purification was performed by washing the DNPs in PBS three times using an Amicon ultra-4 centrifugal filter (MWCO 10 kDa, Millipore, Billerica, MA). The same procedure was used to prepare the INPs by replacing DOX with the same amount of ICG.

### Conjugation of CSA-BP or the SCR to the nanoparticles

Peptides were conjugated to the nanoparticle surface using the EDC/NHS technique (Wen et al., 2017). Briefly, to conjugate the DNPs with the CSA-BP or SCR, the carboxyl groups were activated by adding an activation buffer, EDC and NHS with a DSPE-PEG-COOH:NHS:EDC feed molar ratio of 1:2:2. The EDC/NHS solution was mixed vigorously with the DNPs at room temperature for 30 min, and then, CSA-BP or SCR was added with a feed molar ratio of peptide to DSPE-PEG-COOH of 2:1. The mixture was stirred at room temperature for 2 h and then stored at 4 °C overnight. The products were concentrated again to remove un-conjugated peptides and purified as described above. The CSA-BP- and SCR-conjugated INPs were also prepared using the same method.

### Characterization of the nanoparticles

The surface charge, size, polydispersity, and size distribution of the nanoparticles were determined by dynamic light scattering analysis using a Malvern Zetasizer (Nano ZS, Malvern, UK) at room temperature. The morphology and particle size of the nanoparticles were further analyzed using a transmission electron microscope (TEM, JEM-100CXII, JEOL, Tokyo, Japan) with the negative stain method. Briefly, the samples were soaked onto a copper grid for 5 min, and excess liquid was removed with filter paper. The grid was stained with 2% (w/v) phosphotungstic acid and dried at room temperature before image acquisition.

The encapsulation efficiency (EE) and loading efficiency (LE) of DOX in the nanoparticles were determined as follows: before ultrafiltration, the fresh nanoparticles were isolated from the aqueous suspension medium by ultracentrifugation (34,000×*g*, 30 min) (Optima TM MAX-XP, Beckman, Brea, CA) at 4 °C. The non-entrapped DOX in the supernatant was determined using a fluorescence spectrophotometer (F900, Edinburgh Instruments Ltd., Livingston, UK) with excitation at 480 nm and emission at 593 nm. The EE and LE were calculated as follows:

EE = ((weight of loaded DOX)/(weight of total added drug)) × 100%, and LE = ((weight of loaded DOX)/(total weight of materials)) × 100%.

A dialysis experiment was performed to analyze the DOX release from the different DOX formulations. PBS (pH 7.4) was used as the releasing medium. Different DOX formulations containing 60 μg of DOX were dispersed in 1 mL of PBS and added into the dialysis bags; then, the bags were incubated in 100 mL of PBS at 37 °C under continuous shaking. At predetermined time points, the dialysate was removed to estimate the amount of drug released, while an equal volume of fresh PBS was added to replace the removed volume for further analysis. The DOX concentration in the samples was measured using the fluorescence spectrophotometer as described above.

The concentration of peptides in the supernatant was determined by high-performance liquid chromatography (HPLC, experimental details are in the supporting information). The peptides conjugation efficiency was calculated as follows:

conjugation efficiency = ((total amount – amount in supernatant)/total amount) × 100% (Zhang et al., 2013).

### Cellular uptake of CSA-targeted nanoparticles

The cellular uptake of the different nanoparticles was observed under a fluorescence microscope. First, JEG3 cells grown to 60% confluence in 12-well plates were incubated with free DOX, the DNPs, the SCR-DNPs, or the CSA-DNPs (5 μg of DOX equivalent) for 1 h at 4 °C. Then, the cells were washed with PBS to remove the unbound DOX or nanoparticles and incubated for 30 min at 37 °C to allow internalization of only the surface-bound nanoparticles. Then, the cells were washed three times with PBS, fixed with 4% paraformaldehyde for 15 min and stained with DAPI for 10 min at room temperature. Finally, the cells were visualized under a fluorescence microscope (OLYMPUS IX71, Tokyo, Japan).

For quantitative analysis, the DOX content of the supernatant was detected using a fluorescence spectrophotometer (F900, Edinburgh Instruments Ltd., Livingston, UK) with excitation at 480 nm and emission at 593 nm. Meanwhile, the JEG3 cells were harvested, suspended in 0.5 mL of PBS (0.01 M, pH7.4), and analyzed by flow cytometry (BD, San Jose, CA).

### Subcellular colocalization

The subcellular location of the endocytosed drugs was analyzed using confocal microscopy. Briefly, JEG3 cells were seeded on coverslips and allowed to attach overnight. Then, the medium was replaced with fresh medium supplemented with the CSA-DNPs (containing 5 μg/mL of DOX), 100 nM of LysoTracker Green (Life Technologies, Carlsbad, CA) and 10 μg/mL of Hoechst 33258 (Invitrogen, Carlsbad, CA). The cells were incubated at 37 °C for 30 min and then fixed with 4% paraformaldehyde. Finally, the subcellular localization of the drugs in the cells was observed using confocal laser scanning microscopy (TCS SP5, Leica, Hamburg, Germany).

### *In vitro* cell viability study

Cell viability was evaluated using the Cell Counting Kit-8 (CCK-8, Dojindo, Kumamoto, Japan) assay according to the manufacturer’s instructions. Briefly, JEG3 cells were seeded into 96-well plates at a density of 5000 cells/well. Twenty-four hours later, the medium was replaced with fresh culture medium containing free DOX, the DNPs, the SCR-DNPs, or the CSA-DNPs at DOX concentrations ranging from 0.2 μg/mL to 2 μg/mL. After 24 h, 10 μL of the CCK-8 solution was added to each well, and the plate was incubated at 37 °C for 1 h; then, the absorption at 450 nm was detected using a microplate reader (ThermoFisher, Waltham, MA).

The IC_50_ values were calculated as the concentration of drugs required to inhibit cell growth by 50% compared with the controls and were determined using GraphPad Prism 6.0 software (San Diego, CA).

### Cell apoptosis assay

An Annexin V-FITC/propidium iodide (PI) Apoptosis Detection Kit (Beyotime Institute of Biotechnology, Haimen, China) was used to detect the apoptotic cells according to the manufacturer’s instructions. Briefly, JEG3 cells were seeded in 60-mm dishes at a density of 1 × 10^5^ cell/mL. After 24 h, the cells were treated with or without different nanoparticles (DOX concentration of 0.4 μg/mL). After 24 h, the cells were harvested and resuspended in 0.5 mL of detection buffer containing 10 μL of PI and 5 μL of Annexin V-FITC. Cellular apoptosis was detected and analyzed using flow cytometry (FACSCalibur, BD, San Jose, CA).

### Specific targeting of CSA-NPs *in vivo*

All animal procedures were approved and performed in accordance with the Guidance Suggestions for the Care and Use of Laboratory Animals by the Animal Care and Use Committee from the Shenzhen Institutes of Advanced Technology, Chinese Academy of Sciences. Fluc-GFP-JEG3 cells (10^6^ cells/mouse) were implanted subcutaneously on the right flank of female nude mice (BALB/c background) at 4 weeks of age. When the tumor volume reached approximately 100 mm^3^, CSA-INPs or INPs (1 mg/kg ICG equivalent) were injected intravenously in the tail vein of the mice. Then, the mice were imaged using an IVIS spectrum imaging system (excitation, 710 nm; emission, 840 nm; Perkin-Elmer, Waltham, MA) from 10 min to 48 h.

Forty-eight hours after the nanoparticle injection, the mice were anesthetized with 30% urethane. Fluc-GFP-JEG3 tumors were then collected, and immunofluorescence analysis was performed according to the published procedures (Fan et al., 2014). Briefly, tissues were fixed in 4% paraformaldehyde overnight. After dehydration in 30% sucrose, the tissues were embedded in OCT and frozen. The tumor tissue sections (10 μm) were then stained with DAPI for 10 min at room temperature. Finally, sections were visualized using confocal laser scanning microscopy (TCS SP5, Leica, Hamburg, Germany).

### Tumor models and bioluminescent imaging

For the metastasis model, Fluc-GFP-JEG3 cells (10^6^ cells/mouse) were injected into the lateral tail vein of 4- to 6-week-old female nude mice (BALB/c background). Tumor metastases were evaluated by restricting the region of interest of the bioluminescent image to that of the originally occupied area. To establish the xenograft model, Fluc-GFP-JEG3 cells (10^6^ cells/mouse) were implanted by subcutaneous injection into the right flank region of the mice. The mice were randomly allocated into one of five treatment groups: those treated with the CSA-DNPs, the DNPs, the SCR-DNPs, free DOX (10 mg/kg DOX equivalent per mouse), or PBS alone. Treatments were administered intravenously from day 2 after the xenograft implantation and imaged using the IVIS spectrum imaging system (Perkin-Elmer, Waltham, MA); this process was repeated every other day, with tumor volumes (calculated as (tumor length) × (tumor width)^2^/2) measured and recorded until 18 days after the xenograft.

### Statistical analysis

Statistical values were defined using unpaired Student’s *t*-test, with a *p* value less than .05 considered statistically significant. All statistical analyses were performed using GraphPad Prism 6.0 software (San Diego, CA).

## Results

### Characterization of the CSA-DNPs

**Figure 1.**
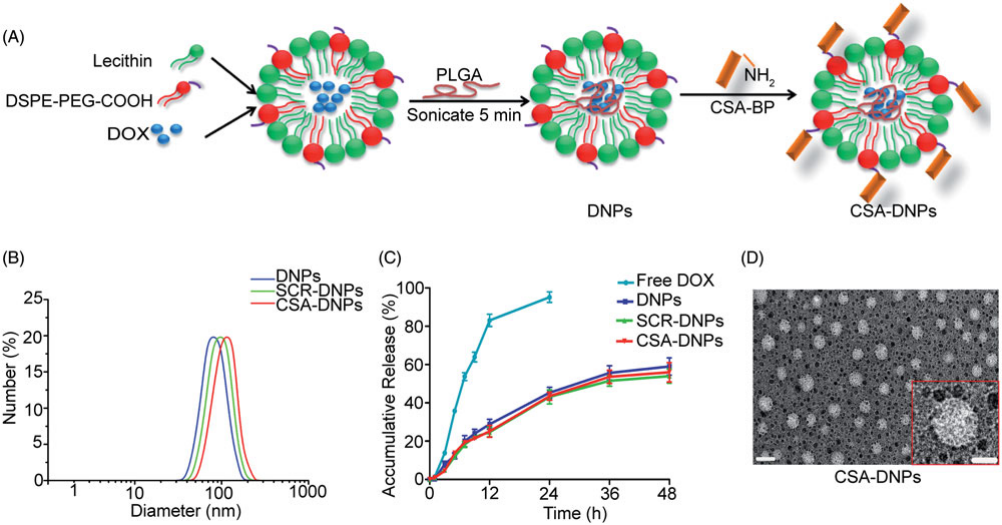
Synthesis and characterization of CSA-DNPs. (A) Schematic illustration of the single-step sonication method to synthesize CSA-DNPs. (B) Size distribution of the different nanoparticles. (C) DOX release profiles of free DOX, DNPs, SCR-DNPs, and CSA-DNPs in PBS. The data are shown as the means ± SD (*n* = 3). (D) TEM image of the CSA-DNPs. The scale bar represents 100 nm (left) and 50 nm (right).

The synthetic procedure is detailed in Figure 1(A). The DNPs were self-assembled from DOX, PLGA, lecithin, and DSPE-PEG-COOH through a single-step sonication method. Bioconjugation was performed between the amino-terminal fragment of the peptide and the carboxyl groups on the surface of the DNPs.

The mean diameters and zeta potentials of the nanoparticles are shown in Table S1 and Figure 1(B). The sizes of the DNPs, SCR-DNPs, and CSA-DNPs were 82.3 ± 4.7 nm, 99.6 ± 4.2 nm, and 109.3 ± 5.9 nm, respectively, indicating that peptide conjugation significantly increased the size of the nanoparticles. Meanwhile, the TEM image of the CSA-DNPs also showed that the particles had generally spherical morphologies (Figure 1(D)). In addition, the peptide used in the present study was not large enough to cause notable changes in the surface charge, thus reducing the influence of surface charge on the nanoparticle behavior *in vitro* and *in vivo* (Alexis et al., 2008). The efficiency of conjugating the CSA-BP and SCR to the DNPs was 49.3 ± 4.4% and 56.7 ± 3.1%, respectively. The EE of DOX for DNPs, SCR-DNPs, and CSA-DNPs was 40.3 ± 1.67%, 38.8 ± 1.83%, and 39.5 ± 1.94%, respectively. The LE of DOX for DNPs, SCR-DNPs, and CSA-DNPs was 6.2 ± 0.74%, 5.3 ± 0.56%, and 5.1 ± 0.42%, respectively. These data indicated that the drug encapsulation ability and loading ability were maintained after peptide decoration.

The *in vitro* release profiles of the DNPs, SCR-DNPs, and CSA-DNPs displayed a similar biphasic pattern (Figure 1(C)), which was characterized by a fast initial release within the first 24 h (approximately 50% release) and a slower and continuous release in the following 48 h (approximately 60% release). These results suggested that conjugating the peptide did not significantly influence the *in vitro* release pattern of DOX.

### Cellular uptake and internalization of CSA-DNPs

**Figure 2.**
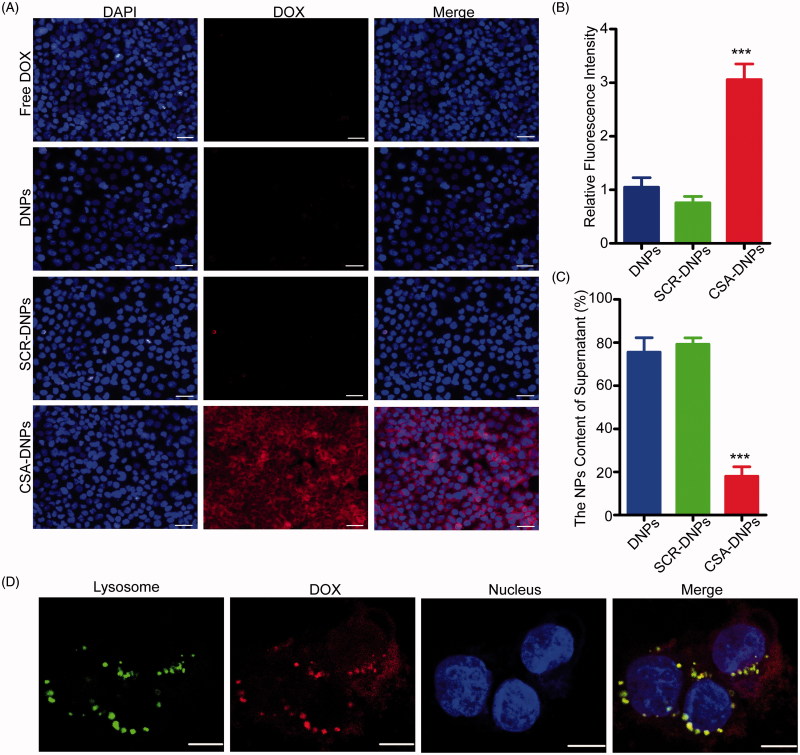
*In vitro* cell uptake and subcellular localization of CSA-DNPs. (A) JEG3 cells analyzed by fluorescence microscopy after a 30-min incubation with 5 μg/mL of different DOX-loaded nanoparticles. The scale bar represents 50 μm. (B) The corresponding quantitative analysis of the cellular fluorescent signal using flow cytometry. Values are expressed as the means ± SD (*n* = 3). (C) Quantitative analysis of the DOX content in different groups using a fluorescence spectrophotometer (data represent the means ± SD, *n* = 3). (D) Subcellular colocalization of the CSA-DNPs in JEG3 cells after a 30-min incubation with the CSA-DNPs. Nuclei were stained with Hoechst 33258, and lysosomes were stained with LysoTracker. Scale bar: 10 μm. ***p < .001 compared with the DNPs.

To test whether conjugating the CSA-BP to the nanocarriers increases the nanoparticle uptake into tumors, JEG3 cells were incubated with the different nanoparticle formulations. Fluorescent microscopy and flow cytometry analysis showed that the nanoparticle uptake into cells was three-fold higher for the CSA-targeted formulation than that for the non-targeted control (Figure 2(A,B)). This result was confirmed by the significantly less DOX that was found in the supernatant of CSA-targeted nanoparticles (Figure 2(C)). In addition, conjugating the SCR did not result in an enhancement of nanoparticle uptake into JEG3 cells, showing the specific role of CSA in the tumor cell uptake of targeted nanoparticles.

The intracellular localization of nanomaterials has been reported to typically involve the lysosomes after the entry of the materials into cells (Zhao et al., 2011, 2016). We further investigated the intracellular localization of CSA-DNPs incubated with cells for 30 min at 37 °C, and then, we removed the unbound nanoparticles. Extensive colocalization between the fluorescent signals of CSA-DNPs (red signals) and lysosomes (green signals) was clearly observed via the yellow dots in the overlaid confocal microscopic image (Figure 2(D)). Based on these data, we conclude that the CSA-targeted nanoparticles are rapidly bound to JEG3 cells and are efficiently internalized into the lysosomes.

### CSA-DNPs display strong growth inhibition and apoptotic effects on JEG3 cells

**Figure 3.**
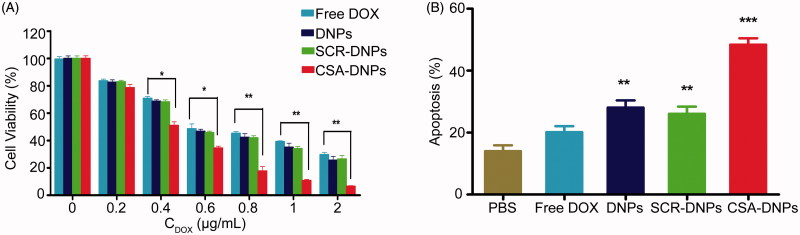
Cell cytotoxicity assay. (A) Quantitative evaluation of the viability of JEG3 cells treated with free DOX and the different nanoparticles for 24 h. *p < .05, **p < .01 compared with free DOX. (B) Apoptosis of JEG3 cells after incubation with or without the different nanoparticles (*n* = 3). **p < .01, ***p < .001 compared with PBS.

Next, the CCK-8 assay was used to evaluate the viability of JEG3 cells after 24 h of treatment with the different nanoparticle formulations. The cell viability profiles of the JEG3 cells exhibited that both the targeted (CSA-DNPs) and non-targeted (DNPs and SCR-DNPs) nanoparticles exerted a dose-dependent cytotoxic effect. At doses of 0.4–2 μg/mL, the CSA-DNPs displayed stronger killing effects on the JEG3 cells than the DNPs or SCR-DNPs (Figure 3(A)). The doses for the cytotoxic effect were determined based on the IC_50_ values of these nanoparticles. The IC_50_ of the CSA-DNPs (0.4 μg/mL) in JEG3 cells was significantly lower than the DNPs (0.69 μg/mL), the SCR-DNPs (0.70 μg/mL), and free DOX (0.90 μg/mL). A quantitative apoptosis assay revealed that after treatment with the CSA-DNPs, the percentage of JEG3 cells in early apoptotic and late apoptosis stages was significantly increased and was 2.4-fold higher than that of free DOX; by contrast, for the cells treated with the DNPs and SCR-DNPs, the number of apoptotic cells was similar, and this value was 1.4- and 1.3-fold higher than that of free DOX, respectively (Figure 3(B)). These data were in agreement with the cellular uptake results, demonstrating that conjugating the DNPs with the CSA-BP leads to strong growth inhibition and apoptosis in JEG3 cells.

### *In vivo* localization of CSA-INPs

**Figure 4.**
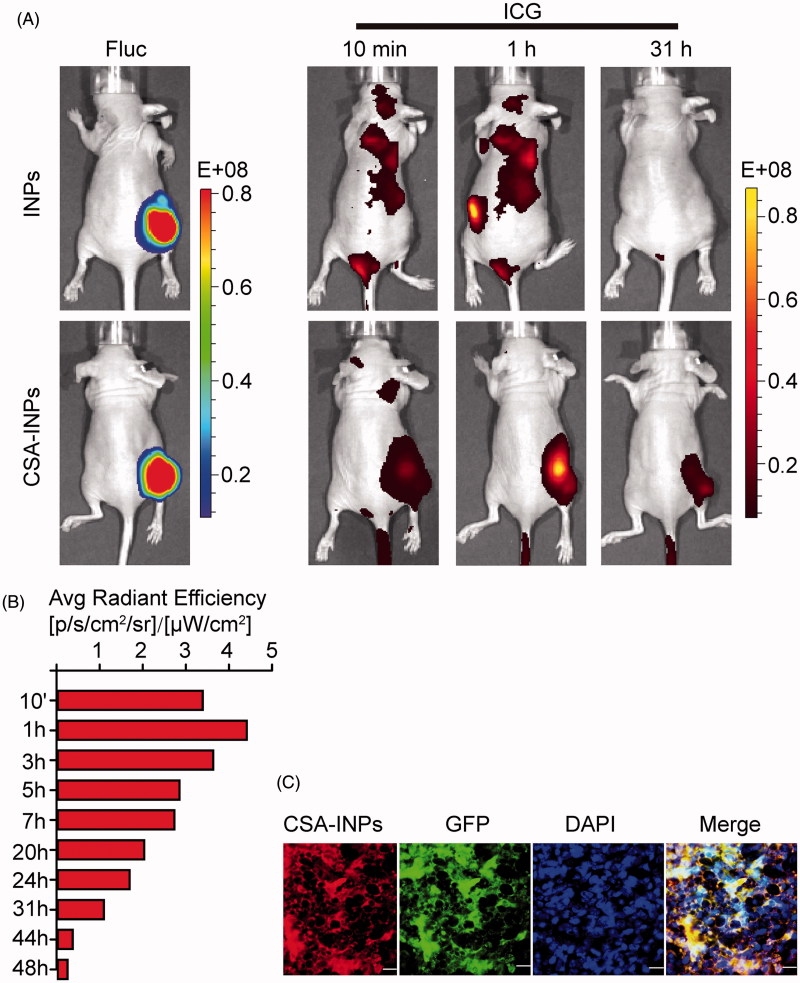
Imaging of CSA-BP-modified INPs *in vivo.* (A) Nude mice bearing Fluc-GFP-JEG3 tumors (left row) were intravenously injected with the CSA-INPs or INPs (1 mg/kg ICG equivalent). Mice were sequentially imaged from 10 min to 48 h using an IVIS spectrum imaging system. (B) Quantification of the IVIS signal from the Fluc-GFP-JEG3 tumors at different time intervals from 10 min to 48 h after the CSA-INPs injection. (C) Immunofluorescence staining of Fluc-GFP-JEG3 tumor tissues 48 h after the CSA-INPs intravenous injection. The sections were imaged using confocal microscopy. Scale bar: 20 μm.

To investigate the tumor targeting of the CSA-NPs *in vivo*, nude mice bearing Fluc-GFP-JEG3 tumors in their right flank were injected intravenously with the CSA-INPs or INPs. Within the tumor region, the ICG signal from the CSA-INPs was rapidly observed after 10 min *in vivo* and could be followed for 48 h after injection. By contrast, there was no ICG signal in the tumor region of mice treated with the INPs (Figure 4(A,B)). Moreover, *ex vivo* immunofluorescence analysis of fixed Fluc-GFP-JEG3 xenografts strongly confirmed the ability of the CSA-INPs to target tumors (Figure 4(C)). These data suggest that the CSA-modified nanoparticles can rapidly target tumors and can be utilized to delivery cytotoxic compounds to tumors *in vivo*.

### CSA-DNPs effectively inhibit primary human choriocarcinoma growth in mice

**Figure 5.**
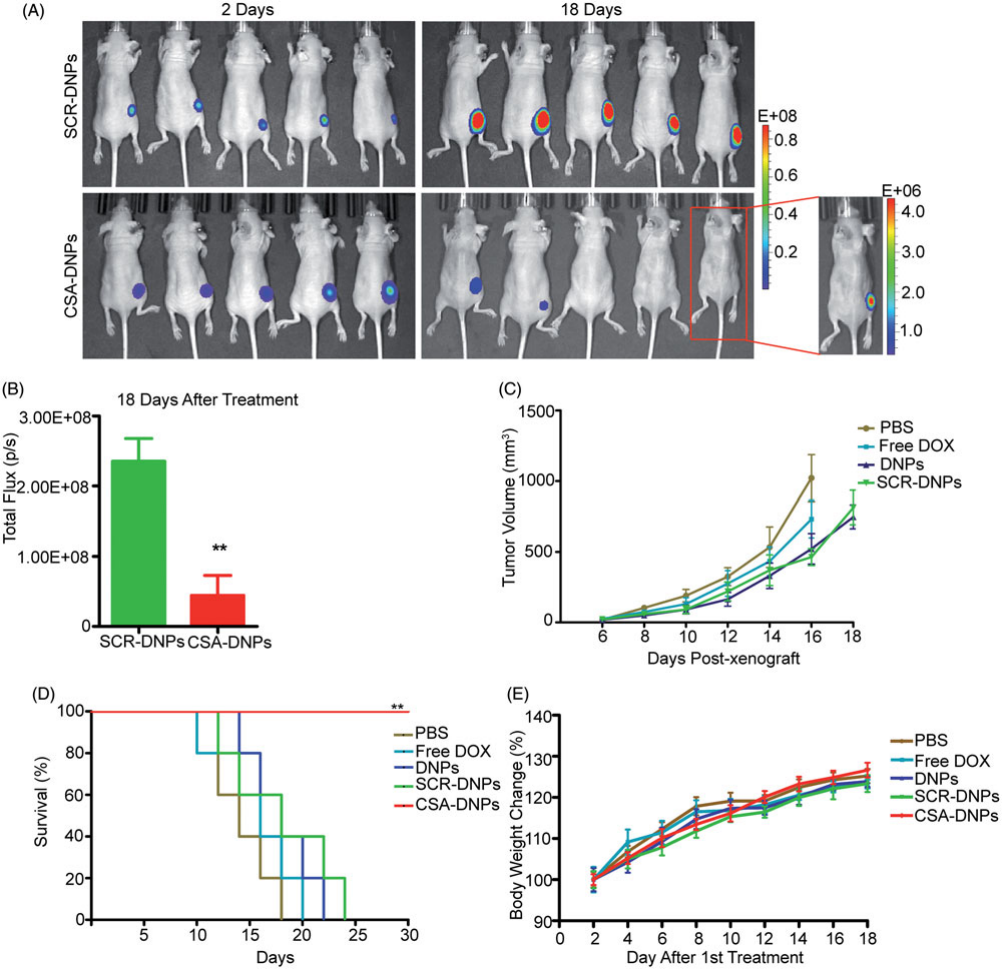
CSA-targeted DNPs inhibited primary tumor growth in mice after systemic administration. (A) IVIS analysis of Fluc-GFP-JEG3 tumor growth in mice receiving different treatments at the indicated time points. At day 2 after the xenograft implantation, the mice (*n* = 5) received an intravenous injection of different nanoparticles (10 mg/kg DOX equivalent); these injections were repeated every other day. (B) Quantification of the total photon flux in mice (from A) at day 18 after the xenograft (data represent the means ± SD, *n* = 5, **p < .01). (C) Caliper measurements of the xenograft volume in mice after the intravenous injection of PBS, free DOX, the DNPs, SCR-DNPs, or CSA-DNPs were repeated every other day (data represent the means ± SD, *n* = 5). No tumors were observed in the CSA-DNP group. (D) Kaplan–Meier’s survival curve comparison of tumor-bearing mice treated with PBS, free DOX, the DNPs, SCR-DNPs, or CSA-DNPs. The mice were sacrificed when they reached their humane endpoint. Statistical significance (**p < .01) was calculated using the Chi^2^ log-rank test of free DOX, the DNPs, SCR-DNPs, and CSA-DNPs compared with PBS. (E) Change in the body weight of mice bearing Fluc-GFP-JEG3 tumors receiving different treatments (means ± SD, *n* = 5). No significant difference among the various treatment groups was observed at any time point.

To evaluate the *in vivo* effect of our CSA-targeted DNPs, Fluc-GFP-JEG3 cells were grafted into the right flank regions of nude mice. Starting at day 2, the mice were treated with the CSA-DNPs, DNPs, SCR-DNPs, free DOX (10 mg/kg DOX equivalent), or PBS alone every other day. The primary tumor burden was monitored using bioluminescent imaging and caliper measurements. Compared with the other groups, the group of mice treated with the CSA-DNPs exhibited a significantly reduced primary tumor burden and no macroscopic tumor growth, whereas the non-targeted SCR-DNPs did not enhance the antitumor effects of DOX (Figure 5(A–C)). Remarkably, the luciferase activity in the Fluc-GFP-JEG3 cells could not be detected in two of the five mice in the CSA-DNP-treated group 18 days after treatment (Figure 5(A)). In addition, all the mice in the PBS group were dead 18 days after tumor implantation (median survival, 14 days). At day 18, a significant increase in survival (100%) was observed in the CSA-DNP-treated mice compared with the mice in the DNPs, SCR-DNPs (40%) and free DOX (20%) groups. All the mice in the CSA-DNP treatment group were alive after the 30-day survival studies (Figure 5(D)). Throughout the entire course of treatment, no significant difference in body weights was observed across the different treatment groups, demonstrating the low toxicity of the targeted CSA-DNPs and non-targeted DNPs or SCR-DNPs administered via intravenous injections (Figure 5(E)). Collectively, these data highlight the effectiveness and safety of the systemic administration of the CSA-DNPs for the inhibition of primary human choriocarcinoma growth in mice.

### CSA-DNPs inhibit human choriocarcinoma metastasis in mice

**Figure 6.**
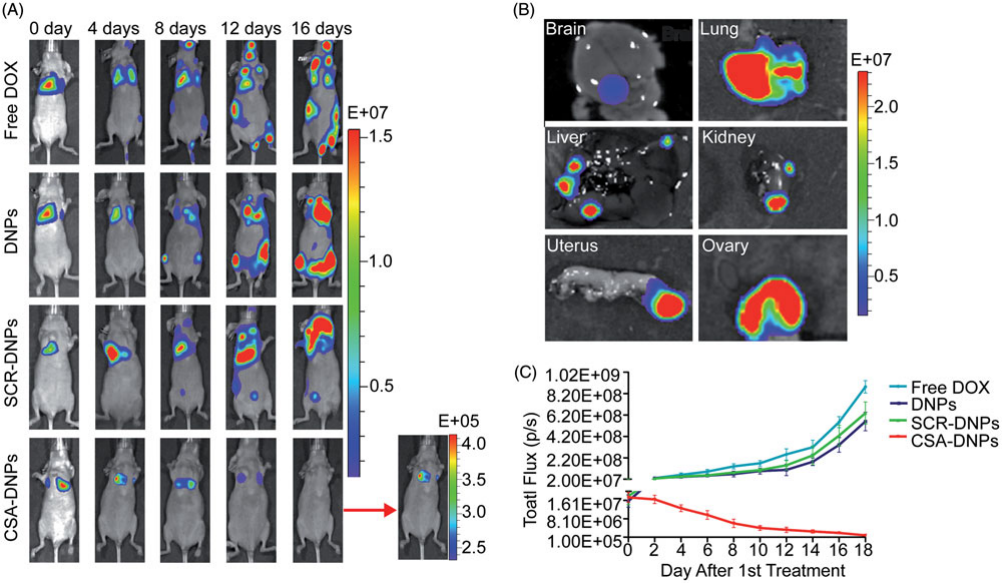
CSA-targeted DNPs inhibited choriocarcinoma metastases in mice. (A) IVIS images of mice after receiving intravenous injections of different nanoparticles. At day 2 after the xenograft implantation, the mice (*n* = 5) received an intravenous injection of free DOX, the DNPs, SCR-DNPs, or CSA-DNPs (10 mg/kg DOX equivalent); these injections were repeated every other day. (B) IVIS images of the organs in nude mice 10 days after receiving an intravenous injection of Fluc-GFP-JEG3 cells. A total of 10^6^ Fluc-GFP-JEG3 cells were intravenously injected via the tail vein, and the mice were analyzed by autopsy and bioluminescent image after 10 days. (C) Quantification of the total photon flux in mice (from A) after injection of the Fluc-GFP-JEG3 cells (data represent the means ± SD, *n* = 5).

We further tested the efficacy of CSA-DNPs in a human choriocarcinoma metastasis mouse model. At day 10 after the Fluc-GFP-JEG3 cells were implanted, mice autopsies and bioluminescent imaging revealed abundant metastatic foci in the lung, liver, kidney, uterus, ovary, and brain of the mice (Figure 6(B)). These data corresponded to the previously reported (Shih, 2007; Cheung et al., 2009) theory that the metastasis of choriocarcinoma occurs via a hematogenous route to many distant organs. Starting on day 2, the animals received intravenous injections of the CSA-DNPs, DNPs, SCR-DNPs, or free DOX (10 mg/kg DOX equivalent) every other day. Compared with the control groups, CSA-DNP treatment resulted in robust inhibition of tumor metastasis (Figure 6(A,C)). Interestingly, treatment with the DNPs or SCR-DNPs also slightly inhibited tumor metastasis compared with treatment with free DOX. This result demonstrated that the nanoparticles could reach tumors via the enhanced permeability and retention (EPR) effect (Chisholm et al., 2009; Manzoor et al., 2012; Guo & Huang, 2014). However, these nanoparticles lacked a specific targeting and binding action to the choriocarcinoma cells, so the efficiency of the anti-cancer effect was lower than when using the targeted CSA-DNPs. Taken together, these results show that the CSA-DNPs could effectively inhibit human choriocarcinoma progression and metastasis.

## Discussion

GTN was the first cancer that exhibited high cure rates when treated with chemotherapy; these cure rates could exceed 98% with fertility retention, which is relatively high compared to other types of cancers (Seckl et al., 2010; Lim et al., 2017). However, GTN still requires high-dose systemic chemo-drug delivery (Seckl et al., 2013), which is harmful to healthy tissues and organs. In this report, we demonstrate the feasibility of using a CSA-BP conjugated to nanoparticles loaded with DOX (CSA-DNPs) to enhance nanoparticle uptake, resulting in the inhibition of cell viability and an increase in the apoptosis of choriocarcinoma in JEG3 cells *in vitro.* Moreover, we demonstrated that CSA-BP-modified INPs rapidly localized to the tumor after being intravenously injected *in vivo* and that the CSA-DNPs efficiently killed tumor cells and strongly inhibited JEG3 cell metastasis in tumor-bearing mice following multiple intravenous injections. Overall, our results indicate that these nanoparticles can be used as an alternate targeting method for choriocarcinoma treatment and as a support for the development of new drug targeting methods for GTN therapies.

Lipid–polymer nanoparticles present a new and promising drug delivery platform with the unique advantages of both liposomes and polymeric nanoparticles, including controllable particle size and surface functionality. These nanoparticles have demonstrated a high drug-loading yield, a sustained drug release profile, and excellent *in vitro* and *in vivo* stability (Zhang et al., 2008; Fang et al., 2010; Zheng et al., 2013). In this study, to generate the lipid–polymer nanoparticles, a single-step sonication method was used to induce self-assembly of the lipids and polymers. Free PLGA (polymers) is dissolved in acetonitrile (water-miscible organic solvent), while lecithin (lipid) and DSPE-PEG-COOH (lipid-PEG conjugates) are dispersed in a 4% ethanol aqueous solution. The PLGA solution is then added to the lecithin aqueous solution dropwise under sonication. When the organic solvent diffuses into the aqueous solution, the PLGA precipitates into small nanoparticles. The lecithin and DSPE-PEG-COOH spontaneously self-assemble on the surface of these PLGA nanoparticles through hydrophobic interactions to reduce the system’s free energy. The hydrophobic tail of lecithin adheres to the hydrophobic PLGA core, and the hydrophilic headgroup of lecithin extends into the external aqueous environment. The DSPE-PEG-COOH participates in the self-assembly process with the DSPE moiety being inserted into the lecithin monolayer and with -PEG-COOH facing outward from the lecithin monolayer to form lipid–polymer nanoparticles.

The drug loading and EE of nanoparticles are crucial for a successful drug delivery system. Increasing the drug content of the nanoparticles with an increased theoretical drug loading may have resulted in increasing the particle size (Dong & Feng, 2004). The particle size plays an important role in determining the drug release behavior of nanocarriers as well as their fate after administration. It has been reported that nanoparticles between 100 and 200 nm have a favorable EPR effect within tumor vasculature (Guo et al., 2011). Moreover, an increase in the drug loading might increase the nanoparticle polydispersity. If the drug concentration is increased to a threshold of the solubilization capacity of the particles, then notable precipitation will occur (Liu et al., 2006). It was reported that the particles were more stable when the drug loading levels were lower (Lin et al., 2003). Meanwhile, the drug loading amount is the key factor for the drug EE. Previous studies showed that a higher drug load resulted in a lower drug EE (Govender et al., 1999; Dong & Feng, 2004). Therefore, in the nanoparticle delivery system, the compatibility between the drug and the particle size is of utmost importance. In the present work, drug loadings of approximately 5% were found to minimize the nanoparticle size and polydispersity, and at this drug loading amount, the fabricated nanoparticles may be more stable and behave more predictably *in vivo*.

Recently, Salanti et al. demonstrated that CSA is exclusively expressed on trophoblastic cells and in most human tumors. They also showed that using the malarial protein VAR2CSA enabled the targeted delivery of anti-cancer compounds into the tumor (Salanti et al., 2015; Seiler et al., 2017). In the present study, a novel trophoblast-targeted nanoparticle system is described for delivering DOX into choriocarcinoma cells using a synthetic CSA-BP derived from VAR2CSA. We showed that the CSA-DNPs could specifically bind to JEG3 choriocarcinoma cells both *in vitro* and *in vivo.* Additionally, the CSA-DNPs were rapidly internalized in 30 min via endocytosis and accumulated in the lysosomes where the acidic lysosome environment and enzyme catalysis facilitated the rapid degradation of the nanoparticles and released the loaded DOX into the cytoplasm. Then, DOX traveled to the nucleus (Cui et al., 2017) and induced JEG3 cell apoptosis. Moreover, the systemic administration of the CSA-DNPs led to an enhanced killing of the primary tumor and inhibition of metastasis. Notably, the delivery of DOX to the tumors *in vivo* using these CSA-targeted nanoparticles minimizes potential toxic side effects. Overall, this improvement resulted in a substantial enhancement of animal survival compared to the control groups. In summary, in line with previous studies (Salanti et al., 2015; Seiler et al., 2017), our data highlight CSA as a marker for a human tumor therapy strategy, and the results indicate that the CSA-BP may be a novel tool for the targeted delivery of drugs to most human cancer cells.

Despite being a recombinant malarial protein, VAR2CSA has been used for the delivery of anti-cancer compounds directly to human tumor cells (Salanti et al., 2015). However, when VAR2CSA is conjugated with nanoparticles, the high molecular weight of the full-length protein may limit its penetration into tumor tissues, and the specificity of the tumor-targeted nanoparticles may be affected by the interaction of the whole protein with normal tissues. The high cost of VAR2CSA also limits its wide use as a ligand for tumor-targeted nanoparticles. Using a synthetic CSA-BP overcomes these problems and has many advantages, such as low molecular weight, small size and low immunogenicity. Importantly, human tumor tissue microarray analysis demonstrated that the CSA-BP could broadly target human cancer cells, including those of hematopoietic, epithelial and mesenchymal origin (unpublished data). Although the partial peptide binding affinity may be lower than that of the intact protein (Resende et al., 2008), CSA-BP conjugated to the nanoparticle surface can form a multivalent bond with receptors because of the multiple copies of CSA-BP on the nanoparticle surface, which in principle, enables increased functional affinity. Thus, the CSA-BP is particularly suitable for the engineering of targeted nanoparticles.

## Conclusions

In summary, the successful application of CSA-BP-modified nanoparticles was demonstrated as an efficient tool for the targeted delivery of chemotherapeutic agents to choriocarcinoma cells and may be a novel platform for targeted human cancer therapy.

## Supplementary Material

IDRD_Fan_et_al_Supplemental_Content.docx

## References

[CIT0001] Alexis F, Pridgen E, Molnar LK, Farokhzad OC. (2008). Factors affecting the clearance and biodistribution of polymeric nanoparticles. Mol Pharm 5:505–15. 18672949 10.1021/mp800051m PMC2663893

[CIT0002] Cheung AN, Zhang HJ, Xue WC, Siu MK. (2009). Pathogenesis of choriocarcinoma: clinical, genetic and stem cell perspectives. Future Oncol 5:217–31. 19284380 10.2217/14796694.5.2.217

[CIT0003] Chisholm EJ, Vassaux G, Martin-Duque P, et al (2009). Cancer-specific transgene expression mediated by systemic injection of nanoparticles. Cancer Res 69:2655–62. 19258509 10.1158/0008-5472.CAN-08-2657

[CIT0004] Clausen TM, Christoffersen S, Dahlbäck M, et al (2012). Structural and functional insight into how the *Plasmodium falciparum* VAR2CSA protein mediates binding to chondroitin sulfate A in placental malaria. J Biol Chem 287:23332–45. 22570492 10.1074/jbc.M112.348839 PMC3390611

[CIT0005] Cui T, Liang JJ, Chen H, et al (2017). Performance of doxorubicin-conjugated gold nanoparticles: regulation of drug location. ACS Appl Mater Interfaces 9:8569–80. 28218512 10.1021/acsami.6b16669

[CIT0006] Dahlbäck M, Jørgensen LM, Nielsen MA, et al (2011). The chondroitin sulfate A-binding site of the VAR2CSA protein involves multiple N-terminal domains. J Biol Chem 286:15908–17. 21398524 10.1074/jbc.M110.191510 PMC3091200

[CIT0007] Dong Y, Feng SS. (2004). Methoxy poly(ethylene glycol)–poly(lactide) (MPEG-PLA) nanoparticles for controlled delivery of anticancer drugs. Biomaterials 25:2843–9. 14962562 10.1016/j.biomaterials.2003.09.055

[CIT0008] Duffy MF, Maier AG, Byrne TJ, et al (2006). VAR2CSA is the principal ligand for chondroitin sulfate A in two allogeneic isolates of *Plasmodium falciparum*. Mol Biochem Parasitol 148:117–24. 16631964 10.1016/j.molbiopara.2006.03.006

[CIT0009] Fan X, Rai A, Kambham N, et al (2014). Endometrial VEGF induces placental sFLT1 and leads to pregnancy complications. J Clin Invest 124:4941–52. 25329693 10.1172/JCI76864 PMC4347223

[CIT0010] Fan X, Ren P, Dhal S, et al (2011). Noninvasive monitoring of placenta-specific transgene expression by bioluminescence imaging. PLoS One 6:e16348. 21283713 10.1371/journal.pone.0016348 PMC3025029

[CIT0011] Fang RH, Aryal S, Hu C-MJ, Zhang L. (2010). Quick synthesis of lipid–polymer hybrid nanoparticles with low polydispersity using a single-step sonication method. Langmuir 26:16958–62. 20961057 10.1021/la103576a

[CIT0012] Ferrari M. (2005). Cancer nanotechnology: opportunities and challenges. Nat Rev Cancer 5:161–71. 15738981 10.1038/nrc1566

[CIT0013] Fried M, Duffy PE. (1996). Adherence of *Plasmodium falciparum* to chondroitin sulfate A in the human placenta. Science 272:1502–4. 8633247 10.1126/science.272.5267.1502

[CIT0014] Frost AS, Sherman JH, Rezaei K, et al (2017). Choriocarcinoma with brain, lung and vaginal metastases successfully treated without brain radiation or intrathecal chemotherapy: a case report. Gynecol Oncol Rep 20:97–9. 28393093 10.1016/j.gore.2017.03.014 PMC5376261

[CIT0015] Gamain B, Trimnell AR, Scheidig C, et al (2005). Identification of multiple chondroitin sulfate A (CSA)-binding domains in the var2CSA gene transcribed in CSA-binding parasites. J Infect Dis 191:1010–13. 15717280 10.1086/428137

[CIT0016] Govender T, Stolnik S, Garnett MC, et al (1999). PLGA nanoparticles prepared by nanoprecipitation: drug loading and release studies of a water soluble drug. J Control Release 57:171–85. 9971898 10.1016/s0168-3659(98)00116-3

[CIT0017] Guo J, Gao X, Su L, et al (2011). Aptamer-functionalized PEG-PLGA nanoparticles for enhanced anti-glioma drug delivery. Biomaterials 32:8010–20. 21788069 10.1016/j.biomaterials.2011.07.004

[CIT0018] Guo S, Huang L. (2014). Nanoparticles containing insoluble drug for cancer therapy. Biotechnol Adv 32:778–88. 24113214 10.1016/j.biotechadv.2013.10.002 PMC3980181

[CIT0019] Kohorn E. (2001). The new FIGO 2000 staging and risk factor scoring system for gestational trophoblastic disease: description and critical assessment. Int J Gynecol Cancer 11:73–7. 10.1046/j.1525-1438.2001.011001073.x 11285037

[CIT0020] Lim W, Yang C, Park S, et al (2017). Inhibitory effects of quercetin on progression of human choriocarcinoma cells are mediated through PI3K/AKT and MAPK signal transduction cascades. J Cell Physiol 232:1428–40. 27714811 10.1002/jcp.25637

[CIT0021] Lin WJ, Juang LW, Lin CC. (2003). Stability and release performance of a series of pegylated copolymeric micelles. Pharm Res 20:668–73. 12739777 10.1023/a:1023215320026

[CIT0022] Liu J, Lee H, Allen C. (2006). Formulation of drugs in block copolymer micelles: drug loading and release. Curr Pharm Des 12:4685–701. 17168772 10.2174/138161206779026263

[CIT0023] Lurain JR. (2011). Gestational trophoblastic disease II: classification and management of gestational trophoblastic neoplasia. Am J Obstetr Gynecol 204:11–18. 10.1016/j.ajog.2010.06.072 20739008

[CIT0024] Manzoor AA, Lindner LH, Landon CD, et al (2012). Overcoming limitations in nanoparticle drug delivery: triggered, intravascular release to improve drug penetration into tumors. Cancer Res 72:5566–75. 22952218 10.1158/0008-5472.CAN-12-1683 PMC3517817

[CIT0025] Nadhan R, Vaman JV, Nirmala C, et al (2017). Insights into dovetailing GTD and cancers. Crit Rev Oncol/Hematol 114:77–90. 10.1016/j.critrevonc.2017.04.001 28477749

[CIT0026] Peer D, Karp JM, Hong S, et al (2007). Nanocarriers as an emerging platform for cancer therapy. Nat Nanotechnol 2:751–60. 18654426 10.1038/nnano.2007.387

[CIT0027] Resende M, Nielsen MA, Dahlbäck M, et al (2008). Identification of glycosaminoglycan binding regions in the *Plasmodium falciparum* encoded placental sequestration ligand, VAR2CSA. Malar J 7:104. 18534039 10.1186/1475-2875-7-104 PMC2430714

[CIT0028] Riehemann K, Schneider SW, Luger TA, et al (2009). Nanomedicine—challenge and perspectives. Angew Chem Int Ed 48:872–97. 10.1002/anie.200802585 PMC4175737 19142939

[CIT0029] Salanti A, Clausen TM, Agerbaek M, et al (2015). Targeting human cancer by a glycosaminoglycan binding malaria protein. Cancer Cell 28:500–14. 26461094 10.1016/j.ccell.2015.09.003 PMC4790448

[CIT0030] Salanti A, Dahlbäck M, Turner L, et al (2004). Evidence for the involvement of VAR2CSA in pregnancy-associated malaria. J Exp Med 200:1197–203. 15520249 10.1084/jem.20041579 PMC2211857

[CIT0031] Salanti A, Staalsoe T, Lavstsen T, et al (2003). Selective upregulation of a single distinctly structured var gene in chondroitin sulphate A‐adhering *Plasmodium falciparum* involved in pregnancy‐associated malaria. Mol Microbiol 49:179–91. 12823820 10.1046/j.1365-2958.2003.03570.x

[CIT0032] Seckl MJ, Sebire NJ, Berkowitz RS. (2010). Gestational trophoblastic disease. Lancet 376:717–29. 20673583 10.1016/S0140-6736(10)60280-2

[CIT0033] Seckl MJ, Sebire NJ, Fisher RA, et al (2013). Gestational trophoblastic disease: ESMO Clinical Practice Guidelines for diagnosis, treatment and follow-up. Ann Oncol 24 Suppl.6:vi39–50. 23999759 10.1093/annonc/mdt345

[CIT0034] Seiler R, Oo HZ, Tortora D, et al (2017). An oncofetal glycosaminoglycan modification provides therapeutic access to cisplatin-resistant bladder cancer. Eur Urol 72:142–50. 28408175 10.1016/j.eururo.2017.03.021

[CIT0035] Shih I-M. (2007). Gestational trophoblastic neoplasia—pathogenesis and potential therapeutic targets. Lancet Oncol 8:642–50. 17613426 10.1016/S1470-2045(07)70204-8

[CIT0036] Taşçı T, Reyen I, Kimyon G, et al (2016). EMA/CO combination chemotherapy in gestational trophoblastic neoplasia: update of our results. Gynecol Obstetr Reprod Med 21:86-92.

[CIT0037] Wen L, Tan Y, Dai S, et al (2017). VEGF-mediated tight junctions pathological fenestration enhances doxorubicin-loaded glycolipid-like nanoparticles traversing BBB for glioblastoma-targeting therapy. Drug Deliv 24:1843–55. 29182025 10.1080/10717544.2017.1386731 PMC8241127

[CIT0038] Zhang B, Sun X, Mei H, et al (2013). LDLR-mediated peptide-22-conjugated nanoparticles for dual-targeting therapy of brain glioma. Biomaterials 34:9171–82. 24008043 10.1016/j.biomaterials.2013.08.039

[CIT0039] Zhang L, Chan JM, Gu FX, et al (2008). Self-assembled lipid–polymer hybrid nanoparticles: a robust drug delivery platform. ACS Nano 2:1696–702. 19206374 10.1021/nn800275r PMC4477795

[CIT0040] Zhao F, Zhao Y, Liu Y, et al (2011). Cellular uptake, intracellular trafficking, and cytotoxicity of nanomaterials. Small 7:1322–37. 21520409 10.1002/smll.201100001

[CIT0041] Zhao P, Zheng M, Luo Z, et al (2016). Oxygen nanocarrier for combined cancer therapy: oxygen-boosted ATP‐responsive chemotherapy with amplified ROS lethality. Adv Healthcare Mater 5:2161–7. 10.1002/adhm.201600121 27253453

[CIT0042] Zhao P, Zheng M, Yue C, et al (2014). Improving drug accumulation and photothermal efficacy in tumor depending on size of ICG loaded lipid–polymer nanoparticles. Biomaterials 35:6037–46. 24776486 10.1016/j.biomaterials.2014.04.019

[CIT0043] Zheng M, Yue C, Ma Y, et al (2013). Single-step assembly of DOX/ICG loaded lipid–polymer nanoparticles for highly effective chemo-photothermal combination therapy. ACS Nano 7:2056–67. 23413798 10.1021/nn400334y

