# Supplementary material for: Targeted delivery of doxorubicin by CSA-binding nanoparticles for choriocarcinoma treatment
Source: Drug Deliv. 2018 Feb 9;25(1):461–71. doi: 10.1080/10717544.2018.1435750 (PMC6058719; doi:10.1080/10717544.2018.1435750)
Supplement: IDRD_Fan_et_al_Supplemental_Content.docx [file IDRD_A_1435750_SM2927.docx]

**Supplemental online material for**

**Targeted delivery of Doxorubicin by CSA-binding Nanoparticles for Choriocarcinoma Treatment**

Baozhen Zhang^a#^, Guogang Cheng^a#^, Mingbin Zheng^b^, Jinyu Han^a^, Baobei Wang^a^, Mengxia Li^a^, Jie Chen^a^, Tianxia Xiao^a^, Jian Zhang^a^, Lintao Cai^b*^, Shoujun Li^c,d*^, and Xiujun Fan^a*^

*^a^Shenzhen Institutes of Advanced Technology, Chinese Academy of Sciences,* *Laboratory for Reproductive Health, Shenzhen, Guangdong, China, 518055.*

*^b^Guangdong Key Laboratory of Nanomedicine, CAS Key Lab for Health Informatics, Institute of Biomedicine and Biotechnology, Shenzhen Institutes of Advanced Technology, Chinese Academy of Sciences, Shenzhen, China, 518055.*

*^c^Guangdong Provincial Key Laboratory of Prevention and Control for Severe Clinical Animal Diseases, Guangzhou, China, 510642.*

*^d^College of Veterinary Medicine,* *South China Agricultural University, Guangzhou, China, 510642.*

*^#^contributed equally to this work*

^*^Corresponding authors: Xiujun Fan, Phone: +86 755 86392360. Fax: +86 755 86392282. E-mail: xj.fan@siat.ac.cn. Lintao Cai, E-mail: lt. [cai@siat.ac.cn](mailto:cai@siat.ac.cn). Shoujun Li, E-mail: shoujunli@scau.edu.cn

Table S1 Characterization of the nanoparticles

|  | Diameter  (nm) | Zeta potential  (mV) | Polydispersity | EE of DOX (%) | LE of DOX (%) | | Conjugation efficiency  (%) |
| --- | --- | --- | --- | --- | --- | --- | --- |
| DNPs | 82.3±4.7 | -20.1±1.32 | 0.127±0.005 | 40.3±1.67 | | 6.2±0.74 | - |
| SCR-DNPs | 99.6±4.2^***^ | -26.0±1.28 | 0.162±0.026 | 38.8±1.83 | | 5.3±0.56 | 56.7±3.1 |
| CSA-DNPs | 109.3±5.9^***^ | -29.9±3.56 | 0.134±0.065 | 39.5±1.94 | | 5.1±0.42 | 49.3±4.4 |

^***^p<0.001 compared with DNPs (n=3)

EE: drug encapsulation efficiency

LE: drug loading efficiency


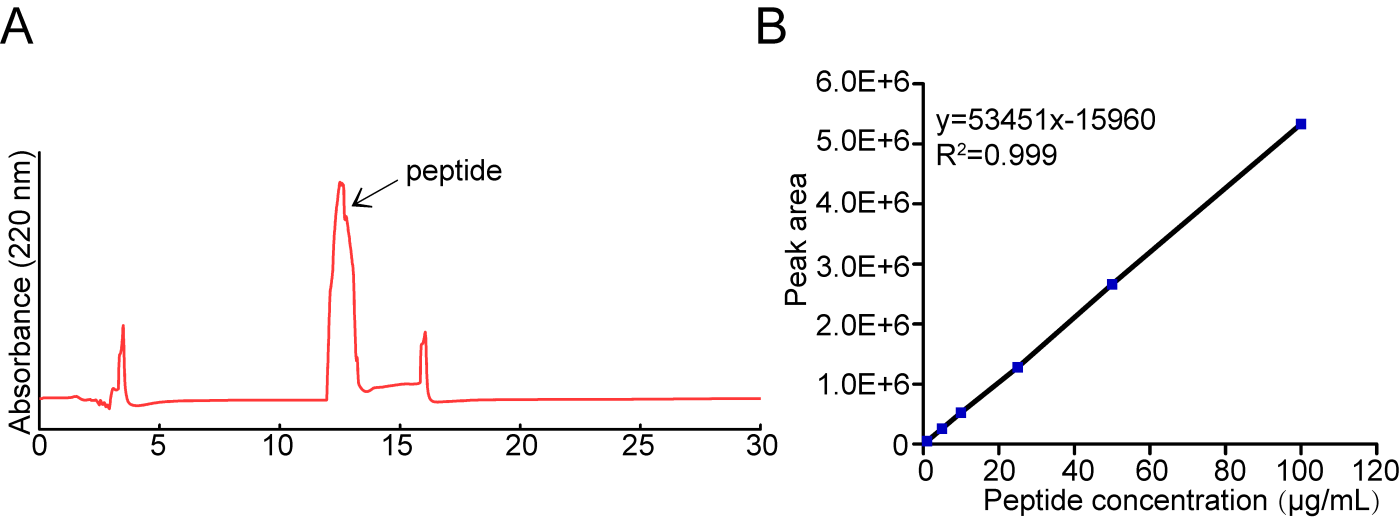


**Figure S1.** **Representative HPLC chromatograms of peptides.** (A) HPLC profile of a peptide with a retention time of 12.5 min. (B) Standard curve for the peptides in the supernatant.

**Supplemental experimental procedures**

***Determination of CSA-BP or SCR conjugation efficiency***

The concentration of peptides in the supernatant was determined by high-performance liquid chromatography (HPLC). HPLC was performed on a reversed phase symmetric C18 column (dimensions, 250×4.6 mm; particle size, 5μm, Waters, MA, USA) with a binary linear gradient elution program. The binary gradient consisted of eluent A (0.1% trifluoroacetic acid in acetonitrile) and eluent B (0.1% trifluoroacetic acid in deionized water). The column was equilibrated with 20% eluent A, and the separation was performed as follows: 0–25 min from 80% to 50% eluent B and 25–30 min from 50% to 0%. A flow rate of 1 mL/min was used with a 10 μL injection volume. The peptides were detected at a UV wavelength of 220 nm (Figure S1).
